# Identifying microRNA/mRNA dysregulations in ovarian cancer

**DOI:** 10.1186/1756-0500-5-164

**Published:** 2012-03-27

**Authors:** Gregory D Miles, Michael Seiler, Lorna Rodriguez, Gunaretnam Rajagopal, Gyan Bhanot

**Affiliations:** 1 The Cancer Institute of New Jersey, New Brunswick, NJ, USA; 2 Department of Bioinformatics and Systems Biology, Boston University, Boston, MA, USA; 3 BioMaPS Institute, Rutgers University, Busch Campus, Piscataway, NJ, USA; 4 Department of Molecular Biology & Biochemistry; Department of Physics, Rutgers University, Piscataway, NJ, USA; 5 Institute for Advanced Study, Simons Center for Systems Biology, Princeton, NJ, USA

## Abstract

**Background:**

MicroRNAs are a class of noncoding RNA molecules that co-regulate the expression of multiple genes via mRNA transcript degradation or translation inhibition. Since they often target entire pathways, they may be better drug targets than genes or proteins. MicroRNAs are known to be dysregulated in many tumours and associated with aggressive or poor prognosis phenotypes. Since they regulate mRNA in a tissue specific manner, their functional mRNA targets are poorly understood. In previous work, we developed a method to identify direct mRNA targets of microRNA using patient matched microRNA/mRNA expression data using an anti-correlation signature. This method, applied to clear cell Renal Cell Carcinoma (ccRCC), revealed many new regulatory pathways compromised in ccRCC. In the present paper, we apply this method to identify dysregulated microRNA/mRNA mechanisms in ovarian cancer using data from The Cancer Genome Atlas (TCGA).

**Methods:**

TCGA Microarray data was normalized and samples whose class labels (tumour or normal) were ambiguous with respect to consensus ensemble K-Means clustering were removed. Significantly anti-correlated and correlated genes/microRNA differentially expressed between tumour and normal samples were identified. TargetScan was used to identify gene targets of microRNA.

**Results:**

We identified novel microRNA/mRNA mechanisms in ovarian cancer. For example, the expression level of RAD51AP1 was found to be strongly anti-correlated with the expression of hsa-miR-140-3p, which was significantly down-regulated in the tumour samples. The anti-correlation signature was present separately in the tumour and normal samples, suggesting a direct causal dysregulation of RAD51AP1 by hsa-miR-140-3p in the ovary. Other pairs of potentially biological relevance include: hsa-miR-145/E2F3, hsa-miR-139-5p/TOP2A, and hsa-miR-133a/GCLC. We also identified sets of positively correlated microRNA/mRNA pairs that are most likely result from indirect regulatory mechanisms.

**Conclusions:**

Our findings identify novel microRNA/mRNA relationships that can be verified experimentally. We identify both generic microRNA/mRNA regulation mechanisms in the ovary as well as specific microRNA/mRNA controls which are turned on or off in ovarian tumours. Our results suggest that the disease process uses specific mechanisms which may be significant for their utility as early detection biomarkers or in the development of microRNA therapies in treating ovarian cancers. The positively correlated microRNA/mRNA pairs suggest the existence of novel regulatory mechanisms that proceed via intermediate states (indirect regulation) in ovarian tumorigenesis.

## Background

Ovarian cancers have a high mortality rate and few treatment options and the failure rate is high [1]. In spite of significant advances in detection and effort to reduce recurrence rates, the five year survival rate has remained relatively unchanged for over 50 years [2].

The Cancer Genome Atlas (TCGA) [3] is a public database which provides multi-modal, patient matched data, including microRNA and mRNA expression levels as well as clinical data (survival, recurrence and treatment), for large cohorts in several cancers, including serous cystadenocarcinoma (the most common type of ovarian cancer). In this paper, we apply a validated method [4] to identify statistically significant microRNA/mRNA regulations which are disrupted in serous cystadenocarcinoma using patient matched data from TCGA. Although mRNA and microRNA dysregulations in ovarian cancer have been identified in various studies [5-15], regulatory mRNA targets of microRNA have not been established. Our analysis proceeds as follows:

We first identify those mRNA or microRNA which can individually and robustly distinguish ovarian cancer samples from normal ovary samples based on expression level. We then identify putative mRNA potentially regulated by the microRNA by matching microRNA/mRNA using seed sequence complementarity from TargetScan http://www.targetscan.org). Finally, we look for a significant correlation/anti-correlation signature between patient matched microRNA/mRNA in tumor samples or normal samples. This procedure allows us to identify microRNA/mRNA regulations which are common (maintained) between tumor and normal tissue as well as microRNA/mRNA regulations that are disrupted in carcinogenesis [4].

Using several statistical tests (Students T-test with FDR correction, unpaired t-test without FDR correction, and Mann-Whitney test with FDR correction), K-Means Clustering [16] and principal component analysis [17], our analysis found 18 microRNA and 49 mRNA which best distinguish tumour from normal. Using seed sequence matches between all putative pairs between these sets (from TargetScan) and Pearson correlation at significance *p *< 0.05 and < 0.1 (one tailed test) in the tumour and normal samples, respectively, we found forty microRNA/mRNA pairs of potential interest, including fifteen pairs anti-correlated across all tumour samples and seven pairs anti-correlated across the normal samples. Within the anti-correlated pairs, one pair: hsa-miR-140-3p/RAD51AP1, was anti-correlated in both tumour and normal samples with opposite expression levels in tumor/normal, implying the dysregulation of a direct microRNA/mRNA mechanism. In addition, we also identified microRNA/mRNA pairs that were correlated or anti-correlated in the tumour samples but not in the normal samples, suggesting a de novo gain of microRNA function in tumours. Also present were microRNA/mRNA regulations present in normal samples but not in tumour samples, indicating a de novo loss of microRNA function in tumours. Interestingly, there are also pairs identified which show positive correlation in both tumour and normal samples, implying the potential existence of indirect pathways or intermediate regulatory mechanisms with a possible role in ovarian tumorigenesis.

## Methods

### TCGA data

The Cancer Genome Atlas (TCGA) is a central bank for multidimensional experimental cancer data, including MicroRNA and cDNA microarray data. These data were obtained from the Data Access Matrix within the TCGA data portal (http://cancergenome.nih.gov/dataportal/data/access/). cDNA microarray experiments measuring mRNA expression were run on the Affymetrix HG-U133A platform (22,277 probesets). microRNA experiments were performed on the Agilent 8 × 15 K Human microRNA-specific microarray V2 platform measuring the expression of 821 microRNAs. Of the 386 TCGA microRNA data samples (378 tumours and 8 normals) and 294 mRNA data samples (including one cell line experiment, which was eliminated), filters were applied such that only samples withboth microRNA/mRNA data were retained. When this was done, we were left with 290 samples (282 tumors, 8 normals). Outliers were then removed as described below, leaving a final cohort of 264 samples (258 tumors, 6 normals).

### Identification of samples with matched microRNA/mRNA data

The TCGA dataset consisted of 386 samples with microRNA expression data (378 tumours and 8 normals) and 294 samples with mRNA expression data (including one cell line experiment). Of these, we retained only samples which had data for both microRNA and mRNA levels. This resulted in a reduced dataset with 290 total samples (282 tumours, 8 normals). Further removal of samples with ambiguous class labels with respect to consensus ensemble clustering reduced this to 258 tumour samples and 6 normals (see below).

### Normalization

The mRNA expression data was normalized using MAS5.0 summarization. We then standard normalized the data: The mean of the summarized intensities for each gene across all experiments was subtracted from the individual intensity for that same gene within a given experiment (per-gene mean subtraction). This value was then divided by the standard deviation of the summarized intensities for each gene across all experiments.

We further normalized the data by performing a 75th percentile shift using GeneSpring GX 11.0 (Agilent Technologies, Inc., Santa Clara, CA, USA). In order to compare our data with previous literature [5], these values were then per-gene mean subtracted and divided by the per-gene standard deviation (similar to the mRNA normalization) to produce normalized measurable microRNA expression values.

### Removal of samples with ambiguous class labels

**Figure 1:**
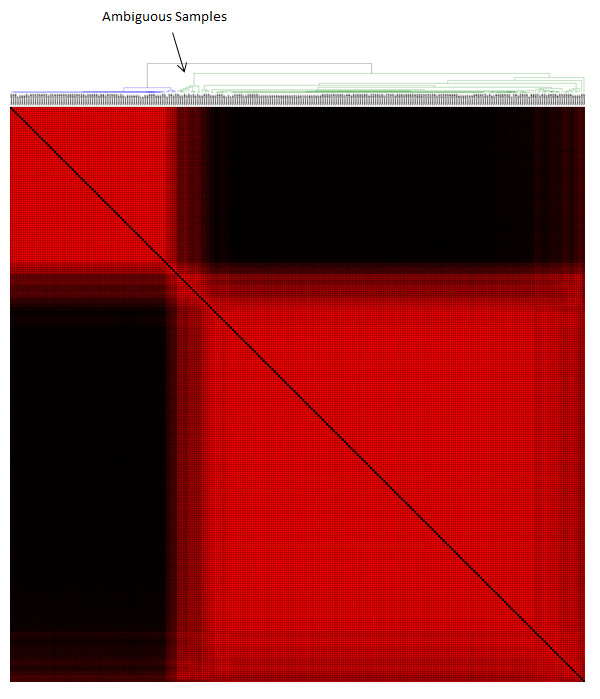
**Identifying ambiguous samples using mRNA levels**. K-means clustering yielded two distinct subclusters which may represent subtypes that will be addressed in the future. The 20 samples which did not cluster well with either group were classified as ambiguous samples and removed.

An ambiguous tumour/normal sample is defined as one that does not robustly cluster with members of its labelled class (tumour or normal). Such samples were identified and eliminated using both mRNA and microRNA data. This was done by using consensus ensemble K-means clustering, using the public software ConsensusCluster (http://code.google.com/p/consensus-cluster/). mRNA expression data for the 290 samples (282 tumours and 8 normals) was clustered via unsupervised K-Means consensus clustering into two classes [18]. Most of the tumour samples clustered in a manner consistent with their labels (tumours with tumours and normal with normal) across the dataset. However, 20 samples clustered in an ambiguous way (sometimes with normal and sometimes with tumours) and were eliminated (Figure 1), leaving 270 samples for to be analysed for their microRNA levels.

We found that microRNA levels varied over a much smaller range than mRNA levels. Hence a similar analysis of the microRNA data required that uninformative microRNA be removed first. We therefore eliminated microRNA whose expression had little or no variation across tumour/normal sample clusters, retaining only those whose expression levels could significantly distinguish tumour samples from normal samples. This was done using three statistical tests:

a) A Students' unpaired t-test with a Benjamini Hochberg FDR multiple testing correction (*p *< 0.05) between the tumour and normal samples within GeneSpring GX 11.0;

b) A Mann-Whitney test with a Benjamini Hochberg FDR multiple testing correction (*p *< 0.05) between the tumour and normal samples within GeneSpring GX 11.0;

c) A Students' unpaired T-test without a multiple testing correction (*p *< 0.05).

**Figure 2:**
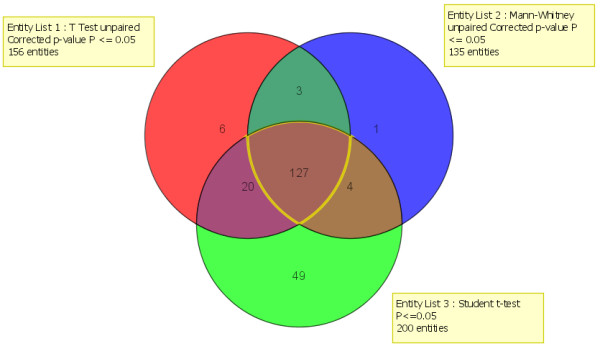
**MicroRNAs that separate tumour from normal**. The intersection of three separate statistical tests yielded 127 microRNA that more significantly differentiate tumour from normal samples. The tests included an unpaired T-test (*p *< 0.05) with a Benjamini Hochberg FDR multiple testing correction and an unpaired Mann-Whitney test (*p *< 0.05) with a Benjamini Hochberg FDR multiple testing correction from within GeneSpring GX 11.0, and a Student's t-test (*p *< 0.05). Data for these 127 microRNA were subsequently used for further clustering of the samples and SNR analysis to uncover the top microRNA which differentiate tumour and normal samples.

Only microRNA that passed all three tests were retained, which resulted in a robust subset of 127 microRNA (Figure 2 and Additional file 1: Table S1) whose expression levels significantly separated tumour samples from normal samples. Consensus ensemble K-means clustering into two clusters using expression levels of these 127 microRNA was performed on the 270 samples that remained after analysis of the mRNA data (see above). This identified six additional samples that did not retain consistent cluster membership across bootstrap samplings of the data. These six samples were also removed.

These two analyses on mRNA and microRNA levels identified our final sample set of 264 samples (258 tumours and 6 normals) which are listed in Additional file 2: Table S2. We note in that our analysis showed that two of the TCGA samples which are labelled as "normal" (TCGA-01-0628-11 and TCGA-01-0631-11) consistently clustered with the tumour samples, suggesting that they might contain a significant contamination from the tumour. These were eliminated from further analysis.

### Identifying optimal microRNA and mRNA to distinguish tumour from normal

**Figure 3:**
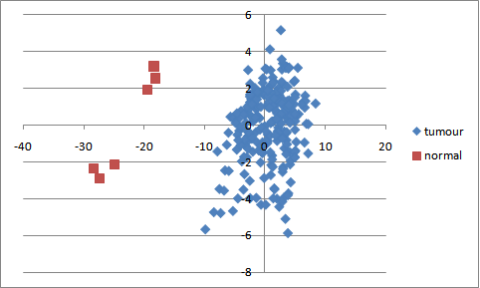
**PCA on robust microRNA data reveals distinct separation**. PCA was executed using only the expression data from the 127 microRNA represented in Figure 1. A clear separation between tumour and normal samples validates the effectiveness of the methods used to generate this microRNA list.

**Figure 4:**
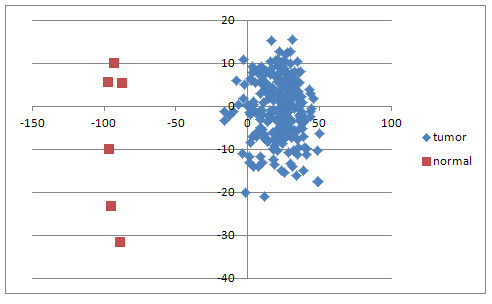
**PCA on mRNA data reveals clear separation**. PCA was executed using gene expression data. A clear separation between the tumour and normal samples indicates the presence of selected genes that robustly distinguish ovarian tumour from the normal samples.

After removal of these ambiguous samples, the ConsensusCluster software was used to perform principal component analysis (PCA) using expression levels of the 127 microRNAs. PCA analysis was performed 43 times, using the six remaining normal samples and six randomly selected tumour samples (without replacement) as input. The first two principal component eigenvectors (PC1 and PC2) from these 43 runs were averaged. Figure 3 shows the PCA plot obtained on projecting the samples onto the two averaged eigenvectors. A robust subset of 18 microRNA was identified as those which appeared most often (35 times out of 43) in these datasets as significantly able to distinguish tumour from normal using a signal-to-noise ratio (SNR) test using SNR > 0.5 as a cutoff. These 18 microRNAs with the most significant values of this eigen-score across the 43 datasets were retained for further analysis. A similar procedure was used for the mRNA, once again validating (using PCA) that the normal samples indeed separate from the tumour samples (Figure 4). Furthermore, upon iterating 43 times once again, the SNR filtering provided 53 genes that were most informative in separating tumour from normal samples in a robust manner, with each gene appearing in at least 20 of the 43 lists generated by ConsensusCluster.

### Identifying mRNA targets of microRNA

TargetScan (http://www.targetscan.org) was used to obtain putative mRNA targets for all 18 microRNAs that best distinguished tumour samples from normals. In the 18 × 53 matrix of microRNA/mRNA pairs, we retained those where the microRNA had a seed sequence in TargetScan which was identified as either 'conserved' or 'poorly conserved'. For each such microRNA/mRNA pair, we computed the Pearson rank correlation function separately within the normal and tumour samples. We retained those which had a *p*-value significance (http://danielsoper.com/statcalc3/calc.aspx?id = 44) < 0.5 for this statistic in tumour samples, and < 0.1 in the normal samples.

## Results

At the time of this study, the TCGA ovarian cancer data set consisted of 282 tumour samples and 8 non-matching normal samples with both microRNA and mRNA expression data available. The microRNA data consists of expression levels for 821 microRNA measured on an Agilent 8 × 15 K Human miRNA-specific microarray platform. The gene expression levels were measured on the Affymetrix GeneChip Human Genome HG-U133A array platform consisting of 22,277 probes. Clinical and survival data were also available for all samples, including: age, days to death (if applicable), days to last follow-up, days to tumour progression, days to tumour recurrence, and age at initial pathologic diagnosis. As described in the Methods section (above), after normalizing, removing ambiguous samples and finding the optimum set of microRNA and mRNA, we were left with 258 tumor samples, 6 normals, 18 microRNA and 53 mRNA.

**Figure 5:**
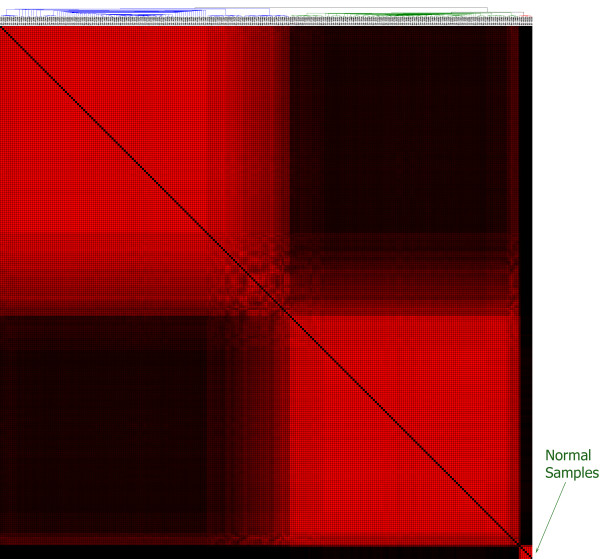
**A separate cluster for normal samples implies significance of 18 microRNA**. We K-means clustered (k = 3) all samples using microRNA expression data exclusively from the 18 microRNA in Table 1. The results are consistent with our expectation that these 18 microRNA best separate the cancerous samples from normal ovarian tissue.

Of the 18 microRNA that robustly distinguished tumours from normals, nine (hsa-miR-183*, hsa-miR-15b*, hsa-miR-15b, hsa-miR-590-5p, hsa-miR-18a, hsa-miR-16, hsa-miR-96, hsa-miR-18b, and dmr_285) were up-regulated and nine (hsa-miR-145*, hsa-miR-143*, hsa-miR-34b*, hsa-miR-140-3p, hsa-miR-145, hsa-miR-139-5p, hsa-miR-34c-3p, hsa-miR-133a, and hsa-miR-34c-5p) were down-regulated in tumour compared to normal. K-means clustering using only these 18 microRNA levels confirmed that these microRNA clearly separate tumour from normal samples (Figure 5).

**Figure 6:**
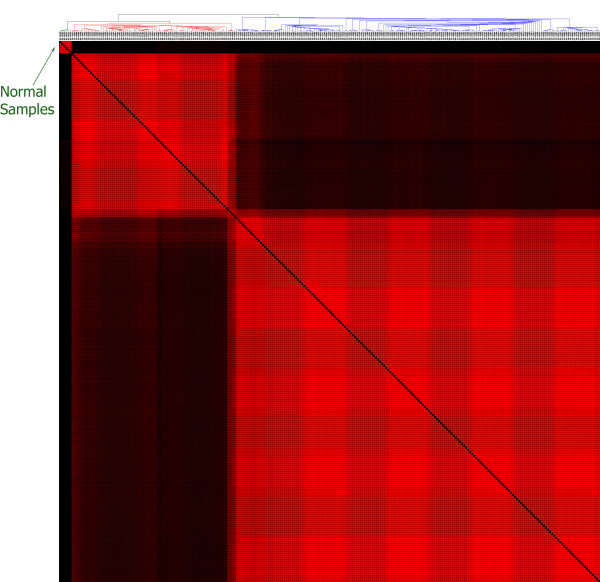
**A distinct cluster containing only normal samples implies significance of 53 mRNAs**. Using SNR, 53 genes were isolated that best separate tumour from normal samples We K-means clustered (k = 3) all samples using expression data from these genes. The above results are consistent with our expectations that these 53 genes separate the cancerous samples from normal ovarian tissue well.

**Figure 7:**
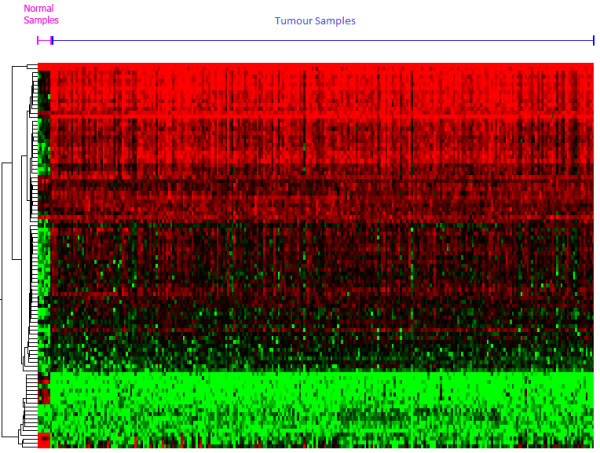
**Heat map of 53 differentially expressed genes in tumours**. Heat map of 53 genes isolated using SNR across 43 iterations. Hierarchical clustering was used to generate the heat map, revealing oncogenic behaviour in the top subcluster and tumour-suppressor activity within the bottom subcluster.

Of the 53 mRNA probes which robustly separated tumour from normal samples, 44 (BUB1, TPX2, CDC25C, ASPM, C1orf112, KIF23, CENPA, HJURP, CCNA2, TTK, CCNB2, C12orf48, BIRC5, RAD51AP1, RACGAP1, MELK, KIFC1, NCAPG, EXO1, KDM2A, EHMT2, DNA2, E2F3, C8orf30A, FAM64A, CORO1B, HEATR3, NCAPH, PSMB2, ERCC6L, KIF15, ESPL1, RANGAP1, KIF11, SCAMP5, NUSAP1, GINS1, ZWINT, ASF1A, an unannotated probe 217205_at, and two probes each for TOP2A and AURKA) were up-regulated and 9 (DNAH3, C6, ADH6, SERPINA6, GCLC, DNAI1, SPARCL1, and two probes for DNAH9) were down-regulated in tumour compared to normal. K-means clustering of using only data from these 53 mRNA confirmed that these mRNA clearly separate tumour from normal samples (Figure 6). We note that the sub-clusters in Figure 6 suggests the presence of two (or more) distinct disease subtypes within the tumour samples. We will further explore these potential disease subclasses in a subsequent paper. Figure 7 shows a heat map of the data projected on the 53 genes that best separate tumour samples from normal samples, and shows that they define a highly accurate signature for this separation.

Of the 18 × 53 possible microRNA/mRNA pairs, 69 pairs showed seed sequence complementarity and conservation in TargetScan. The Pearson Rank test at *p *< 0.5 identified 21 significant pairs (Table 1) in the tumour samples. Of these, fifteen pairs (including nine mRNA) showed a significant anti-correlation signal while six showed a significant positive correlation signal. A similar analysis of the normal samples, at *p *< 0.1, identified nineteen significant pairs (Table 2) of which seven (with six distinct mRNA) exhibited anti-correlation and twelve were positively correlated.

**Table 1:** Anti-correlated and positively correlated mRNA/microRNA pairs in the tumour and/or the normal samples

microRNA	mRNA	normal	tumour	MicroRNA Regulation in tumour vs. normal	Gene Regulation in tumour vs. normal	*P*-value
hsa-miR-140-3p	RAD51AP1	-	-	Down	Up	0.002
hsa-miR-96	KIF23	none	-	Up	Up	0.006
hsa-miR-96	BIRC5	none	-	Up	Up	0.003
hsa-miR-140-3p	RACGAP1	none	-	Down	Up	0.012
hsa-miR-139-5p	RACGAP1	none	-	Down	Up	0.012
hsa-miR-133a	GCLC	none	-	Down	Down	0.023
hsa-miR-16	E2F3	none	-	Up	Up	0.027
hsa-miR-140-3p	C8ORF30A	none	-	Down	Up	0.022
hsa-miR-15b	C8ORF30A	none	-	Up	Up	0.011
hsa-miR-16	C8ORF30A	none	-	Up	Up	0.001
hsa-miR-16	SCAMP5	none	-	Up	Up	3.7E-4
hsa-miR-18a	DNAI1	none	-	Up	Down	0.038
hsa-miR-18b	DNAI1	none	-	Up	Down	0.014
hsa-miR-590-5p	E2F3	+	-	Up	Up	0.020
hsa-miR-34c-5p	E2F3	+	-	Down	Up	0.028
hsa-miR-16	CDC25C	+	+	Up	Up	0.016
hsa-miR-18a	GINS1	+	+	Up	Up	0.0
hsa-miR-18b	GINS1	+	+	Up	Up	0.0
hsa-miR-18a	RAD51AP1	none	+	Up	Up	6.1E-06
hsa-miR-18b	RAD51AP1	none	+	Up	Up	6.2E-07
hsa-miR-15b	RACGAP1	none	+	Up	Up	0.009

a) This Table lists the 21 mRNA/microRNA regulatory pairs found in ovarian tumour samples. The microRNA shown are also differentially expressed in tumour samples compared with normal, exhibit seed sequence complementarity in the gene shown, and are significantly anti-correlated (red) or positively correlated (blue) with the corresponding mRNA in the tumour samples. The third and fourth columns show the type of correlation (if present) within normal and tumour samples respectively. A "+" symbol represents a positive correlation, while "-" represents an anti-correlation, and "none" represents no significant correlation across the samples

**Table 2:** Anti-correlated and positively correlated mRNA/microRNA pairs in the tumour and/or the normal samples

microRNA	mRNA	normal	tumour	MicroRNA Regulation in tumour vs. normal	Gene Regulation in tumour vs. normal	*P*-value
hsa-miR-140-3p	RAD51AP1	-	-	Down	Up	0.021
hsa-miR-139-5p	DNAH9	-	None	Down	Down	0.036
hsa-miR-139-5p	TOP2A	-	None	Down	Up	0.009
hsa-miR-140-3p	GCLC	-	None	Down	Down	0.036
hsa-miR-145	E2F3	-	none	Down	Up	0.009
hsa-miR-139-5p	E2F3	-	none	Down	Up	0.009
hsa-miR-96	FAM64A	-	none	Up	Up	0.002
hsa-miR-590-5p	E2F3	+	-	Up	Up	0.036
hsa-miR-34c-5p	E2F3	+	-	Down	Up	0.036
hsa-miR-16	CDC25C	+	+	Up	Up	0.0
hsa-miR-18a	GINS1	+	+	Up	Up	0.036
hsa-miR-18b	GINS1	+	+	Up	Up	0.036
hsa-miR-590-5p	TOP2A	+	none	Up	Up	0.036
hsa-miR-590-5p	RAD51AP1	+	none	Up	Up	0.021
hsa-miR-18a	RAD51AP1	+	none	Up	Up	0.021
hsa-miR-18a	GCLC	+	none	Up	Down	0.036
hsa-miR-15b	E2F3	+	none	Up	Up	0.021
hsa-miR-18a	NUSAP1	+	none	Up	Up	0.036
hsa-miR-590-5p	GINS1	+	none	Up	Up	0.036

b) This Table lists the 19 mRNA/microRNA regulatory pairs found in normal ovary samples. The microRNA shown are also differentially expressed in tumour samples compared with normal, exhibit seed sequence complementarity in the gene shown, and are significantly anti-correlated (red) or positively correlated (blue) with the corresponding mRNA in the normal samples. The third and fourth columns show the type of correlation (if present) within normal and tumour samples respectively. A "+" symbol represents a positive correlation, while "-" represents an anti-correlation, and "none" represents no significant correlation across the samples

We found that of the fifteen microRNA/mRNA pairs anti-correlated in tumour, one (hsa-miR-140-3p/RAD51AP1) was anti-correlated and fourteen either showed no correlation or were positively correlated across the normal samples. Furthermore, of the six positively correlated pairs in tumours, three showed no correlation and three showed positive correlation within the normal samples, implying a potential indirect mechanism of ovarian tumorigenesis. Of the seven pairs anti-correlated in normal samples, all (except miR-140-3p/RAD51AP1) showed no correlation across the tumour samples, implying a potential de novo loss in microRNA function in tumours. Of the twelve pairs which are positively correlated in normal samples, two show anti-correlation within the tumour samples, implying a potential de novo gain in microRNA function in tumours. The remaining ten pairs, which are positively correlated in normal samples, showed either a positive correlation or no correlation in tumour samples. These are of interest because they suggest some novel indirect mechanisms in ovarian tumorigenesis.

## Discussion

The methods we applied find potential functional relationships that are good candidates for experimental validation. Many of these microRNA/mRNA pairs impact critical cellular functions that are frequently dysregulated in cancer. For instance, RAD51AP1, a gene that functions in double-stranded DNA repair, is also positively expressed in cervical cancer [19] and breast cancer cell lines [20], cancers that exhibit high expression homogeneity with malignant ovarian carcinomas. Our analysis shows that RAD51AP1 is also up-regulated in ovarian cancers. Furthermore, hsa-mir-140-3p, a potential regulator of RAD51AP1 according to our anti-correlation analysis, has been previously shown to be down-regulated in ovarian cancer [20], a finding which we confirm. Interestingly, the anti-correlated relationship exhibited with this pair is also present in the normal samples. This suggests that if RAD51AP1 is acting as an oncogene in ovarian tumours then targeting hsa-mir-140-3p may be a way to target RAD51AP1.

The E2F transcription factor 3 (E2F3), a gene that is up-regulated in serous ovarian carcinomas, regulates crucial cell cycle and tumour suppressor genes [21-30]. We find that this gene is over-expressed in ovarian tumours. Moreover, hsa-miR-145, a microRNA that is highly down-regulated in ovarian cancer [5,8,9], shows significant expression anti-correlation with E2F3 in normal samples, indicating potential transcriptional repression by hsa-miR-145 in normal cells but loss of this function in tumours. Another potential regulator of E2F3, hsa-miR-139-5p, is also predicted to target Topoisomerase IIα (TOP2A), a gene encoding an enzyme which is involved in altering DNA topology, including chromosome condensation, chromatid separation and the relief of torsional stress occurring in transcription and replication. TOP2A has also been shown to be overexpressed in ovarian tumours [31] and is currently a common target in ovarian cancer clinical trials. Glutamate-cysteine ligase (GCLC), an enzyme of glutathione synthesis predicted to be regulated by hsa-miR-133a in tumours (in a de novo gain of microRNA function) and by hsa-miR-140-3p (in a de novo loss of microRNA function), has been shown to be an anti-apoptotic gene that is positively expressed in ovarian cancer cell lines [32,33].

In addition to many of the genes above which have normal microRNA regulation disrupted by a loss of microRNA function (Table 1) in tumours, several genes regulated by microRNAs with de novo gains in function exist as well. Baculoviral inhibitor of apoptosis repeat-containing 5 (BIRC5), or Survivin, is a protein predicted to be regulated by hsa-miR-96 that inhibits caspase activation [34] leading to negative regulation of apoptosis [35]. Furthermore, increased presence of this protein has been shown to decrease apoptosis in cisplatin-sensitive ovarian carcinoma cells [35], a finding that is consistent with the up-regulation of this gene within the TCGA tumour data set. Additionally, another gene found to be up-regulated in tumours in our dataset, Rac GTPase-activating protein 1 (RACGAP1) is an enzyme whose overexpression has also been associated with serous ovarian carcinoma [30].

We also found six positively correlated microRNA/mRNA pairs with matching seed sequences in tumour and eleven pairs in normal. We reasoned that a likely explanation for this lies in an intermediate regulatory mechanism that is probably anti-correlated with the microRNA of interest. Consequently, it is important to note that some of the positively correlated genes/microRNA pairs also impact important cellular functions frequently dysregulated in cancer. Also noteworthy is that three of the pairs show positive correlation in both the tumour and normal samples. This finding suggests that while regulation of gene expression by these microRNA is indirect, physiological differences between tumour and normal samples is directly dependent on the changes in expression by these microRNA and the downstream effects they have on the target mRNA they are paired with. For instance, nucleolar and spindle associated protein 1 (NUSAP1), is a nucleolar-spindle-associated protein that plays a role in spindle microtubule organization [36] that is positively correlated with hsa-miR-18a in normal but not in tumour. It has also been previously shown to be positively regulated in cervical carcinoma [19,36]. Another gene of potential interest is cell division cycle 25 homolog C (CDC25C), a critical gene involved in cell division that dephosphorylates cyclin B-bound CDC2 (CDK1) and triggers entry into mitosis [37]. It might also have a role in suppressing p53-induced growth arrest and has been shown to be overexpressed in ovarian cancer cell lines [27,38]. Finally, GINS complex subunit 1 (GINS1), which was shown to be significantly positively correlated with hsa-miR-18a and hsa-miR-18b in both tumour and normal samples (and positively correlated with hsa-miR590-5p in normal samples), represents a key component of the GINS complex that is essential for initiation of DNA replication and is positively regulated in serous ovarian carcinoma [39]. Interestingly, hsa-miR-18a, which possesses a significant positive correlation with GINS1 (along with NUSAP1) has shown to be highly up-regulated in serous ovarian carcinoma both previously [10] and within our analysis.

Hsa-miR-16, mentioned previously to be anti-correlated in tumours with several potential mRNA targets including E2F3, C8ORF30A, and SCAMP5, is significantly positively correlated with CDC25C. Should seed sequence complementarity and conservation between these positively correlated pairs be purely coincidental, the relationships between the pairs of positively correlated entities suggest an elaborate mechanism by which these oncogenes/tumour suppressors operate. Further wet-lab validation may show this to be a significant ovarian cancer biomarker. This finding may further elucidate a broader cancer pathway involving CDC25C and some of its target tumour suppressor and cell cycle genes.

## Conclusions

Using microRNA/mRNA expression data for 258 tumor samples and six normal samples from TCGA, we have uncovered forty mRNA/microRNA regulation which meet the following criteria: a) These pairs have seed sequence complementarity b) They are able to clearly differentiate ovarian tumours from normal ovary by their expression levels; c) They exhibit a strong anti-correlation or correlation signature within either the tumour samples, the normal samples, or both.

The novelty of our finding is that we have significantly reduced the space of possible dysregulated microRNA/mRNA pairs (based on seed sequence complementarity alone) to a robust subset which may potential represent functional relationships. Such a possibility can be tested experimentally on ovarian cancer cell lines knock down and/or knock in assays. If in addition, some of these dysregulations are associated with survival pathways for the tumor or with resistance to therapy, it opens up the possibility of patient specific targeted therapy.

### Availability of supporting data

All expression data is available for download at The Cancer Genome Atlas Data Portal (http://tcga-data.nci.nih.gov/tcga/tcgaHome2.jsp).

## Competing interests

The authors declare that they have no competing interests.

## Authors' contributions

GM obtained the data, performed most of the analysis, implemented the methods, interpreted the results, and wrote the manuscript, MS performed portions of the analysis, suggested alternative interpretations/analyses to perform, GB, LR, GR helped design and supervise the study. All authors have read and approved the final manuscript. The authors declare that they have no conflict of interest with any of the contents of this manuscript.

## Supplementary Material

Additional file 1 **Table S1 MicroRNAs separating tumour from normal samples**. 127 microRNAs are significantly differentially expressed and robustly separate tumour samples from normal samples. Click here for file

Additional file 2 **Table S2 Samples used for analysis**. This table identifies the 258 tumor samples and 6 normal samples with matched microRNA/mRNA data from the TCGA sample set which were retained for analysis ambiguous samples were removed using consensus ensemble clustering (see Methods for details). Click here for file
